# Impaired Vitamin D Signaling in T Cells From a Family With Hereditary Vitamin D Resistant Rickets

**DOI:** 10.3389/fimmu.2021.684015

**Published:** 2021-05-19

**Authors:** Fatima A. H. Al-Jaberi, Martin Kongsbak-Wismann, Alejandro Aguayo-Orozco, Nicolai Krogh, Terkild B. Buus, Daniel V. Lopez, Anna K. O. Rode, Eva Gravesen, Klaus Olgaard, Søren Brunak, Anders Woetmann, Niels Ødum, Charlotte M. Bonefeld, Carsten Geisler

**Affiliations:** ^1^ The LEO Foundation Skin Immunology Research Center, Department of Immunology and Microbiology, University of Copenhagen, Faculty of Health and Medical Sciences, Copenhagen, Denmark; ^2^ Novo Nordisk Foundation Center for Protein Research, University of Copenhagen, Faculty of Health and Medical Sciences, Copenhagen, Denmark; ^3^ RNA and Gene Medicine Program, Department of Cellular and Molecular Medicine, University of Copenhagen, Faculty of Health and Medical Sciences, Copenhagen, Denmark; ^4^ Department of Nephrology, University of Copenhagen, Rigshospitalet and Faculty of Health and Medical Sciences, Copenhagen, Denmark

**Keywords:** vitamin D, vitamin D receptor, HVDRR, T cells, vitamin A

## Abstract

The active form of vitamin D, 1,25-dihydroxyvitamin D_3_ (1,25(OH)_2_D_3_), mediates its immunomodulatory effects by binding to the vitamin D receptor (VDR). Here, we describe a new point mutation in the DNA-binding domain of the VDR and its consequences for 1,25(OH)_2_D_3_ signaling in T cells from heterozygous and homozygous carriers of the mutation. The mutation did not affect the overall structure or the ability of the VDR to bind 1,25(OH)_2_D_3_ and the retinoid X receptor. However, the subcellular localization of the VDR was strongly affected and the transcriptional activity was abolished by the mutation. In heterozygous carriers of the mutation, 1,25(OH)_2_D_3_-induced gene regulation was reduced by ~ 50% indicating that the expression level of wild-type VDR determines 1,25(OH)_2_D_3_ responsiveness in T cells. We show that vitamin D-mediated suppression of vitamin A-induced gene regulation depends on an intact ability of the VDR to bind DNA. Furthermore, we demonstrate that vitamin A inhibits 1,25(OH)_2_D_3_-induced translocation of the VDR to the nucleus and 1,25(OH)_2_D_3_-induced up-regulation of CYP24A1. Taken together, this study unravels novel aspects of vitamin D signaling and function of the VDR in human T cells.

## Introduction

Both vitamin D and vitamin A have fundamental effect on immune responses (1–4). The active forms of the vitamins, 1,25-dihydroxyvitamin D_3_ (1,25(OH)_2_D_3_) and retinoic acid (RA), act by binding to the vitamin D receptor (VDR) and retinoic acid receptor (RAR), respectively. VDR and RAR belong to the nuclear receptor family class II. They consist of several functional domains, which include a ligand- and a DNA-binding domain (DBD), and they share wide sequence homology (5, 6). After binding of the active vitamins, both receptors bind the retinoic X receptor (RXR) to form VDR-RXR and RAR-RXR heterodimers, respectively. The heterodimers accumulate in the cell nucleus, where they regulate gene transcription by binding to specific DNA sequences called vitamin D response elements (VDRE) and RA response elements (RARE). Transcriptional regulation can be either negative or positive depending on the specific VDRE and RARE sequences and the recruitment of co-activators or co-repressors (7, 8). It has been suggested that 1,25(OH)_2_D_3_ and RA might antagonize the effects of each other by inducing a competition between VDR and RAR for binding to RXR (9, 10). Furthermore, it has been suggested that VDR-RXR dimers can bind RARE and thereby inhibit RA-induced gene regulation (11–13). Accordingly, it has been reported that 1,25(OH)_2_D_3_ inhibits RA-induced up-regulation of the gut-homing receptors integrin α4β7 and CC chemokine receptor (CCR) 9 in T cells (14). The mechanisms behind the interplay between vitamin D and A signaling in T cells are unknown and still need to be determined.

Hereditary vitamin D resistant rickets (HVDRR) is a very rare autosomal, recessive disease caused by mutations in the VDR (9, 15, 16). HVDRR is characterized by early-onset rickets, normal serum levels of 25(OH)D_3_ and increased levels of 1,25(OH)_2_D_3_ often associated with hypocalcemia, secondary hyperparathyroidism, elevated alkaline phosphatase and variable hypophosphatemia (9, 15, 16). Cells from HVDRR patients with mutations in the DBD of the VDR are unresponsive to 1,25(OH)_2_D_3_ and they therefore constitute a unique possibility to study the consequences of abolished 1,25(OH)_2_D_3_ signaling in T cell responses. In addition, studies of heterozygous carriers of a mutated VDR might further contribute to the knowledge of the immunomodulatory role of the VDR and vitamin D signaling in T cells.

In the present study, we describe a family with a new point mutation in the DBD of the VDR resulting in HVDRR. We characterize the mutation and how it affects the subcellular localization and function of the VDR in human T cells.

## Material and Methods

### Case Report and Test Subjects

The patient was born in 1992 in Iraq to consanguineous (first cousins) parents as their fifth child. Her four elder siblings and the parents were apparently healthy, whereas the patient developed rickets and alopecia within the first year after her birth. Two cousins of the patient had similar symptoms as the patient. Test results from her time in Iraq are not available, but according to her parents, the patient was successfully treated orally with 1-α-hydroxycholecalciferol (alfacalcidol), calcium and fish oil, although the alopecia persisted. The treatment was terminated when she was three years old. In 1998, the family moved to Denmark, and in 1999, the patient was referred to hospital due to muscle and bone pains, short stature and alopecia. At the physical examination, the patient appeared normal except for alopecia and short stature with a height and weight below the 3% percentile (**Supplementary Figure 1**). No swelling of the epiphysis or costochondral junctions or obvious bending of the bones of the arms and legs was seen. She had normal serum levels of calcium, phosphate, alkaline phosphatase and parathyroid hormone. However, at several measurements, the level of serum 1,25(OH)_2_D was highly elevated between 320 - 388 pM (normal range 51 – 177 pM). The levels of 24,25(OH)_2_D were low in the normal range between 0.51 – 0.81 nM (normal range 0.37 – 13.4 nM) and the levels of 25(OH)D normal between 39 – 60 nM (normal range 26 – 150 nM). Radiological bone examination of her left hand when she was 7 years and 3 months old showed a bone age of 5 years and 9 months. Based on her case history, the high levels of serum 1,25(OH)_2_D and the delayed bone age it was concluded that the patient suffered from HVDRR, and treatment with calcium in the form of a minimum of 500 ml milk equal to 600 mg calcium per day and calcitriol (rocaltrol) 0.5 µg twice daily was initiated. The following years, she was regularly seen in the outpatient clinic. She often complained of bone pains in the arms and legs but otherwise she was doing well, and she partially catched up for her low stature, body weight and delayed bone age (**Supplementary Figure 1**). The serum levels of 1,25(OH)_2_D were permanently elevated but she kept on having normal serum levels of calcium, phosphate, alkaline phosphatase and parathyroid hormone. At the time of inclusion in the study, the patient was 27 years old, her heterozygous siblings between 31 and 37 years old, her parents 63 and 65 years old, and the control group (5 women and 8 men) between 20 and 62 years old. The study was approved by The Committees of Biomedical Research Ethics for the Capital Region in Denmark (H-170409222). Written consent were obtained from all test subjects in accordance with the Declarations of Helsinki principles for research involving human subjects.

### CD4^+^ T Cell Purification and Activation

Mononuclear cells from donor blood were isolated by Lymphoprep (Axis Shield from Oslo, Norway) density gradient centrifugation by using SepMate™ tubes (85450, Stemcell Technologies, Canada). Naïve CD4**^+^** T cells were isolated from the mononuclear cell fraction by using EasySep Human Naïve CD4**^+^** T cell Enrichment Kit (19155, Stemcell Technologies) according to the manufacturer’s directions. Purified naive CD4**^+^** T cells were cultured at a concentration of 1 x 10^6^ cells/ml in serum-free X-VIVO 15 medium (BE02-060F, Lonza, Verviers, Belgium) for 72 h in flat-bottomed 24-well tissue culture plates (142475, Nunc). For activation of naive CD4**^+^** T cells, Dynabeads Human T-Activator CD3/CD28 beads (111.31D, Life Technologies, Norway, Oslo) were added to the cell cultures in a ratio of 2 beads per 5 cells. Furthermore, for some experiments, the culture medium was supplemented with 25(OH)D_3_, 1,25(OH)_2_D_3_ or RA.

### Chemicals and Antibodies

25(OH)D_3_ (BML-DM-100-0001) and 1,25(OH)_2_D_3_ (BML-DM200-0050) were from Enzo Life Sciences, Inc., Ann Arbor, MI. Stock solutions of 2.5 mM 25(OH)D_3_ and 2.4 mM 1,25(OH)_2_D_3_ were prepared in anhydrous (>99.5%) ethanol. 1,25(OH)_2_D_3_ concentrations in cell culture supernatants were measured by using the 1,25-Dihydroxy Vitamin D EIA kit (AC-62F1, IDS, Tyne and Wear, UK) according to the instruction of the manufacturer. The RA 9-cis-retinoic acid (R4643) was from Sigma-Aldrich. Stock solutions of 3.325 mM RA were prepared in anhydrous (<99.5%) ethanol. Primary antibodies used in Western blotting analyses included anti-VDR (D-6, Santa Cruz Biotechnology) and anti-GAPDH from (Ab9485, Abcam, Cambridge, MA).

### *In Silico* Analysis

In order to analyze possible effects of the R80W mutation on the native structure of the VDR, we performed *in silico* analysis. The crystal structure of VDR bound to a DNA VDRE sequence was obtained from the protein data bank PDB entry 1KB2 (https://www.rcsb.org/structure/1KB2) and analyzed using MODELLER (17).

### Genetic Analysis

PCR amplification of the open reading frame of the VDR mRNA using primers; 5’-ATGGAGGCAATGGCGGC (forward) and 5’-TCATGGCTGAGGTCTCAAGG (reverse) was performed on random hexamer generated cDNA using SuperScript III (ThermoFisher Scientific) from 10 ng whole cell RNA isolated from T cells. Sequencing of the PCR products, using the same primers as above, revealed a single nucleotide substitution at position 238 (C238T) in the open reading frame of the VDR mRNA compared to the control and reference sequence (hg38). The mutation was confirmed on genomic DNA from T cells isolated from blood. 1 x 10^6^ cells were lysed in 100 µl nuclease-free H_2_O for 10 min on ice, adjusted to proteinase K buffer (10 mM Tris-HCL and 0.5 mM EDTA, pH 8.0) and treated with 2 µl of 20 mg/ml Proteinase K solution (ThermoFisher Scientific) for 60 min at 55°C. Subsequently, genomic DNA was extracted using 1 vol of PCI, pH 8.0 followed by 1 vol of chloroform and ethanol precipitated in the presence of 300 mM KAc and 15 µg glycogen. Exon 3 containing the mutation was amplified by PCR and sequenced using primers; 5’-GGCAGGCGAAGCATGAAGC (forward) and 5’-TCACACTCCTTCATCATGCCG (reverse). All family members and controls were genotyped as described above.

### RT-qPCR

mRNA levels for various targets were measured by RT-qPCR. Following cell isolation, cells were lysed in TRI reagent (T9424, Sigma Aldrich) and mixed with phase separation reagent 1-bromo-3-chloropropane (B9673, Sigma Aldrich). The RNA phase was isolated and mixed with isopropanol supplemented with glycogen for RNA precipitation (10814-010, Invitrogen). The RNA pellet was then washed in RNase free 75% ethanol 3 times. cDNA was synthesized from quantified RNA using High-Capacity RNA-to-cDNA™ Kit (4387406, Applied Biosystems) according to manufacturer’s instructions. For RT-qPCR, 12.5 ng cDNA was mixed with TaqMan^®^ Universal Master Mix II with Uracil-N glycosylase and the target primers. We used the following primers from Applied Biosystems: VDR (Hs01045840_m1), CYP24A1 (Hs00167999_m1), CYP27B1 (Hs01096154_m1), GAPDH (Hs02786624_g1), CD38 (Hs1120071_m1), α4 (ITGA4, Hs00168433_m1) and β7 (ITGB7, Hs01565750_m1) and CCR9 (Hs01890924_s1). The plate-based detection instrument LightCycler ^®^ 480 II from Roche was used for real-time PCR amplification.

### RNA-Sequencing Analysis

RNA was isolated as described above. Total RNA sequencing libraries (Ribominus) was constructed and sequenced on an Illumina HiSeq2500. RNA-seq reads was pseudo-aligned to the human transcriptome (GRCh38, rel96) using kallisto (v0.46.2) (18). Estimated counts were length corrected, and regularized log2 values were calculated using the tximport (v1.14) and DESeq2 (v1.22) R packages, respectively, and mitochondrial genes were filtered out. Genes that were at least 1.5-fold different between CD4^+^ T cells activated in the presence or absence of 25(OH)_2_D_3_ were considered differentially regulated.

### Western Blotting Analysis

For Western blotting analysis, cells were lysed in lysis buffer (50 mM Tris base, pH 7.5, 150 mM NaCl and 1 mM Mg2Cl) supplemented with 1% (vol/vol) Triton X-100, 1 x Protease/phosphatase inhibition cocktail (5872S, Cell Signaling Technologies) and separated by electrophoresis through NuPAGE™ 10% BisTris gels (NP0302BOX or NP0301BOX, Life Technologies). For specific detection of proteins in the cytosol and the nucleus, reagents from the NE-PER Nuclear and Cytoplasmic Extraction Reagent kit (78833, Thermo Fisher Scientific) were used according to the manufacturer’s instructions with few modifications as previously described (19, 20). Presence and absence of GAPDH was used as marker for the cytosolic and the nuclear fractions, respectively. Proteins were transferred to nitrocellulose membranes (LC2001, Life Technologies) and visualized with primary antibodies and HRP-conjugated rabbit anti-mouse Ig (P0260, DAKO) or HRP-conjugated swine anti-rabbit Ig (P0399, DAKO) with ECL luminescence reagent (RPN2232, Sigma Aldrich) on a ChemiDocTM MP Imaging System (Bio Rad) and subsequently analyzed using the software ImageLab.

### ELISA

Cytokine concentrations were determined by ELISA using Ready-Set-Go kits (IL-13, 88-7439-22; IFN-γ, 88-7316-88) according to the manufacturer’s instruction as previously described (21).

### Quantification and Statistical Analysis

Quantification of bands from Western blotting analysis was performed using the programs ImageLab (BioRad) and Fiji. Statistical analysis and graphical representation of all data was performed using Prism 7 (GraphPad Soft-ware) and Adobe Illustrator CS6 (Adobe Systems Incorporated). Mann Whitney U tests were used when comparing responses in control cells with responses in mutated cells. Paired Student’s t-tests were used when comparing responses in the same group of cells treated in two different ways. p < 0.05 was considered statistically significant. Data are represented as mean values with one standard deviation (SD) as indicated in each figure legend. The number of donors (n) in each experiment is indicated in the figure legends.

## Results

### The R80W Mutation Abolishes the Transcriptional Activity Without Affecting the Structure or Expression of VDR^R80W^

**Figure 1:**
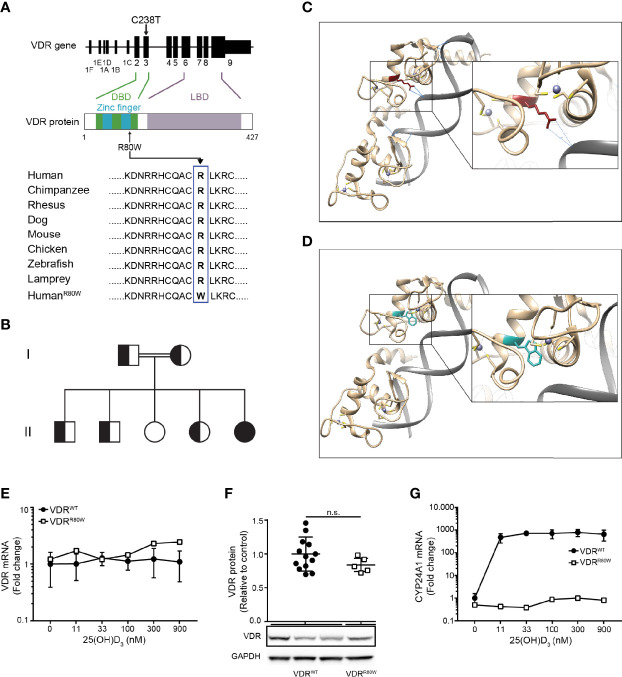
The R80W mutation abolishes the transcriptional activity without affecting the structure or expression of VDR^R80W^ **(A)** Localization of the C238T mutation to exon 3 in the *VDR* gene resulting in the R80W in the C-terminal part of the second zinc finger in the DBD of the VDR. The amino acids surrounding the R80 in the VDR from different species with the R80W at the bottom. **(B)** Family tree where circles represent females, squares represent males, homozygous VDR^R80W^ marked as filled black, heterozygous VDR^WT/R80W^ marked as filled black and white, and homozygous VDR^WT^ marked as filled white. **(C, D)** *In silico* structure of the zinc fingers of the VDR interacting with DNA. **(C)** VDR^WT^ with the R80 in red and with the hydrogen bonds between R80 and the DNA backbone indicated as blue lines. **(D)** The same region of VDR^R80W^ with the W80 in blue. **(E)** VDR mRNA, **(F)** VDR protein and **(G)** CYP24A1 mRNA in VDR^WT^ (black circles) and VDR^R80W^ (white squares) CD4^+^ T cells activated in the presence of the indicated concentration of 25(OH)D_3_. **(E–G)** The expression levels are given as fold change normalized to the average expression level of VDR^WT^ in control cells activated in the absence of 25(OH)D_3_. **(E)** Mean ± SD (VDR^WT^ n = 6; VDR^R80W^ n = 1). **(F)** Relative VDR protein expression as determined by the density of the VDR bands from Western blotting analysis of VDR^WT^ and VDR^R80W^. The upper panel gives the density of VDR^R80W^ (white squares) normalized to the average density of VDR^WT^ (black circles) from control cells. The lower panel shows one representative Western blotting analysis out of five independent experiments of VDR and GAPDH (loading control) from three controls and the patient. Mean + SD (VDR^WT^ n = 13; VDR^R80W^ n = 1 repeated 5 times).** (G)** Mean ± SD (VDR^WT^ n = 3; VDR^R80W^ n = 1). n.s. means not significant.

To identify the mutation in the VDR of the HVDRR patient, we sequenced the *VDR* gene and discovered a cytosine to thymine mutation at position 238 (C238T) (**Figure 1A** and **Supplementary Figure 2A**). This is a previously undescribed missense mutation located in exon 3 of the *VDR* gene that causes exchange of arginine (R) to tryptophan (W) at position 80 (R80W) in the second zinc-finger of the DBD (**Figure 1A**). R80 and the amino acids surrounding it are extremely conserved through evolution (**Figure 1A**) (16). We subsequently sequenced the family members and found that the parents and three of the siblings were heterozygous (VDR^WT/R80W^) carriers of the mutation and one sibling was homozygote for the wild-type VDR (VDR^WT^) (**Figure 1B** and **Supplementary Figure 2A**).

In order to investigate possible structural implications of the VDR^R80W^ mutation, we next performed *in silico* analysis. As previously shown (22), we found that R80 directly forms hydrogen bonds with the backbone of the DNA in DNA-VDR^WT^ complexes (**Figure 1C**). However, these hydrogen bonds were no longer present in DNA-VDR^R80W^ complexes (**Figure 1D**). The R80W mutation did not appear to disturb the native structure of the VDR as most W80 rotamers were allowed without affecting the local structure of the VDR (**Supplementary Figure 2B**). Thus, the *in silico* analysis indicated that the mutation did not affect the structural integrity or physicochemical properties of VDR^R80W^ besides the interaction with DNA.

Normally, human T cells strongly up-regulate VDR expression following activation (20, 23). To determine whether the VDR^R80W^ mutation affected VDR expression, we activated CD4^+^ T cells from the patient and controls in the presence of 0 – 900 nM 25(OH)D_3_ and measured VDR mRNA expression by RT-qPCR. We found equal amounts of VDR mRNA in T cells from the patient and controls indicating that the VDR^R80W^ mutation did not affect VDR transcription (**Figure 1E**). The presence of 25(OH)D_3_ did not affect VDR transcription in neither the controls nor the patient. We next determined the expression of the VDR^WT^ and VDR^R80W^ at the protein level by Western blotting analyses of lysates obtained from activated CD4^+^ T cells from the patient and controls. Activated T cells from the patient clearly expressed the mutated VDR^R80W^, although we consistently found a slightly, but not significantly, reduced expression level of VDR^R80W^ compared to VDR^WT^ (**Figure 1F)**.

The vitamin D 24-hydroxylase CYP24A1 is one of the most strongly vitamin D-induced genes in cells expressing the VDR (7). To study the transcriptional activity of the mutated VDR^R80W^, we measured CYP24A1 mRNA expression in activated CD4^+^ T cells from the patient and controls by RT-qPCR. As expected, CYP24A1 was strongly up-regulated in control cells activated in the presence of 25(OH)D_3_ (**Figure 1G**). In contrast, cells from the patient were unaffected by 25(OH)D_3_ and did not up-regulate CYP24A1 even at the highest concentration of 25(OH)D_3_ tested (**Figure 1G**).

**Figure 2:**
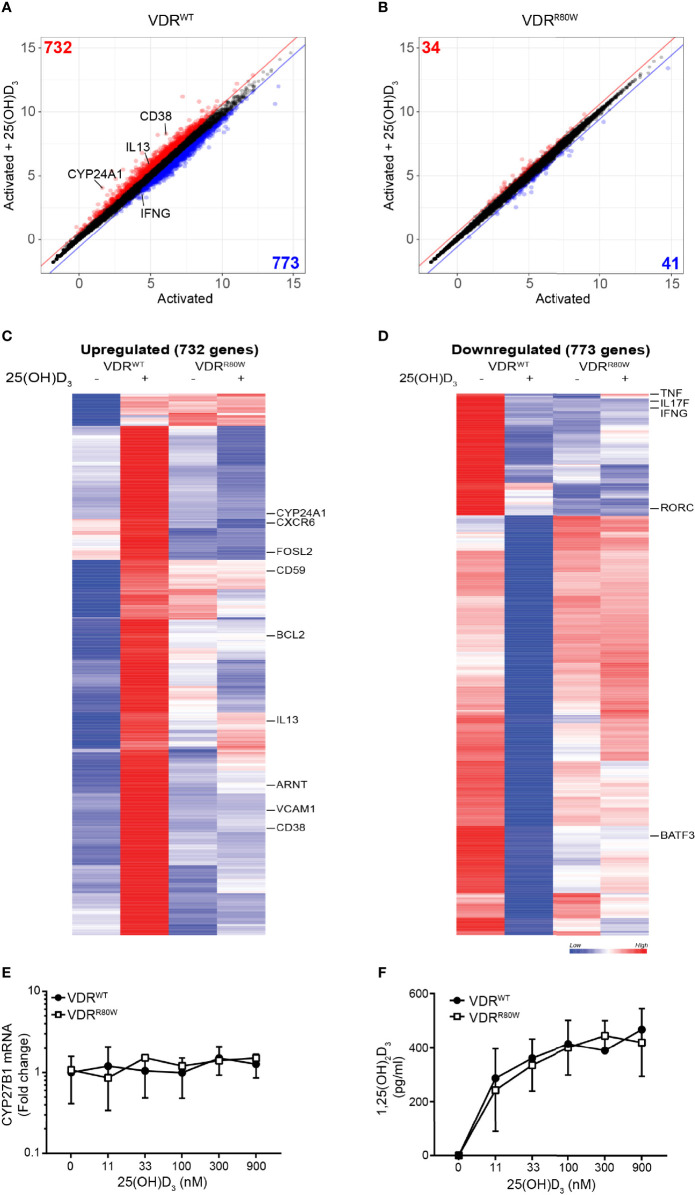
The R80W mutation abolishes normal 1,25(OH)_2_D_3_-mediated gene up- and down-regulation Regularized log2 normalized gene expression in **(A)** VDR^WT^ and **(B)** VDR^R80W^ CD4^+^ T cells activated in the presence or absence of 25(OH)D_3_. The red and blue lines indicate the 1.5-fold threshold for up- and down-regulated genes, respectively. Genes that are at least 1.5-fold up- or down-regulated by 25(OH)D_3_ are colored red or blue, respectively, and the number of genes up- or down-regulated by 25(OH)D_3_ is indicated in the respective corners. Heatmap of genes up- **(C)** and down-regulated **(D)** by 25(OH)D_3_ in VDR^WT^ CD4^+^ T cells. Selected genes are indicated to the right of the heatmaps. **(E)** CYB27B1 mRNA and **(F)** 1,25(OH)_2_D_3_ production in VDR^WT^ (black circles) and VDR^R80W^ (white squares) CD4^+^ T cells activated in the presence of the indicated concentration of 25(OH)D_3_. **(E)** The expression levels are given as fold change normalized to the average expression level of VDR^WT^ in control cells activated in the absence of 25(OH)D_3_. Mean ± SD (VDR^WT^ n = 6; VDR^R80W^ n = 1). **(F)** Mean ± SD (VDR^WT^ n = 6; VDR^R80W^ n = 1).

To determine whether the impaired transcriptional activity of the VDR^R80W^ affected a wider set of genes, we measured mRNA expression in CD4^+^ T cells from the patient and controls activated in the absence and presence of 25(OH)D_3_ by RNA-seq. We identified 732 and 773 genes up- and down-regulated at least 1.5 fold by vitamin D in T cells from the control subjects, respectively. We found that approximately 95% these genes was un-affected by vitamin D in the HVDRR patient (**Figures 2A–D** and Supplementary list of vitamin D-regulated genes). Thus, the mutated VDR had lost its transcriptional activity for the vast majority of genes supporting that the ability of VDR^R80W^ to bind DNA was strongly affected.

To rule out that the missing response to 25(OH)D_3_ was caused by a defect in expression of the 25(OH)D-1-α hydroxylase CYP27B1 in the cells of the patient, we concomitantly measured CYP27B1 mRNA expression in the activated T cells and production of 1,25(OH)_2_D_3_ in the cell culture supernatants. We found that T cells from the patient and controls expressed similar levels of CYP27B1, and that the expression was not affected by 25(OH)D_3_ (**Figure 2E**). In line with this, equal amounts of 1,25(OH)_2_D_3_ were produced by T cells from the patient and the controls (**Figure 2F**).

### The R80W Mutation Does Not Affect Binding to 1,25(OH)_2_D_3_ and RXR but Significantly Affects the Subcellular Localization of VDR^R80W^

**Figure 3:**
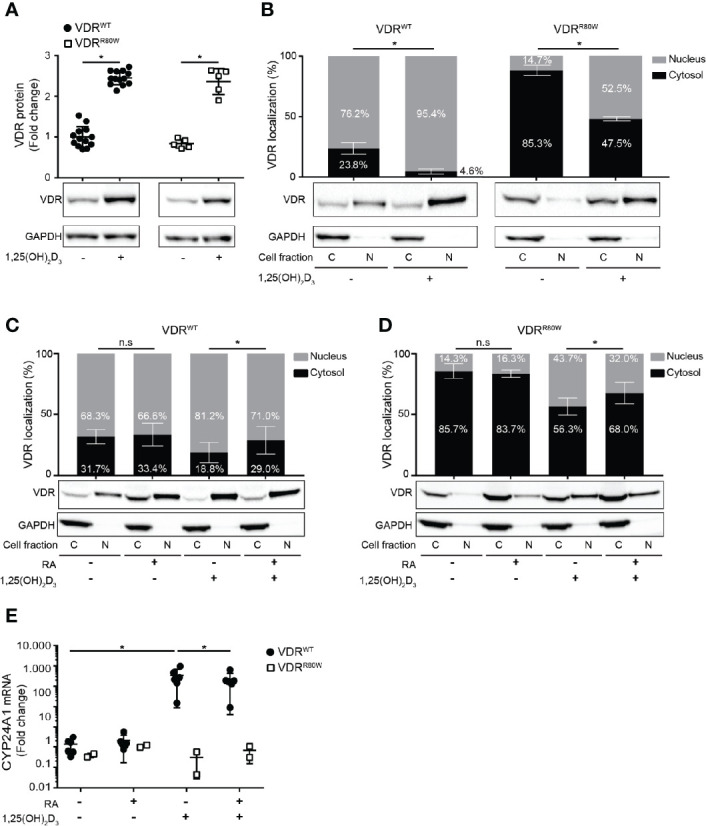
The R80W mutation does not affect binding to 1,25(OH)_2_D_3_ and RXR but significantly affects the subcellular localization of VDR^R80W^ **(A)** Relative VDR protein expression as determined by the density of the VDR bands from Western blotting analysis of VDR^WT^ and VDR^R80W^ CD4^+^ T cells activated in the absence (-) or presence (+) of 1,25(OH)_2_D_3_ (10 nM). The upper panel gives the density of VDR^WT^ (black circles) and VDR^R80W^ (white squares) normalized to the average density of the VDR^WT^ bands from control cells activated in the absence of 1,25(OH)_2_D_3_. The lower panel shows one representative Western blotting analysis out of five independent experiments of VDR and GAPDH (loading control) from control and patient cells. Mean ± SD (VDR^WT^ n = 13; VDR^R80W^ n = 1 repeated 5 times; *p < 0.05). **(B–D)** Subcellular localization of VDR^WT^ and VDR^R80W^ in CD4^+^ T cells activated in **(B)** the absence (-) or presence (+) of 1,25(OH)_2_D_3_ (10 nM) and in **(C, D)** the absence (-) or presence (+) of RA (1 µM) and 1,25(OH)_2_D_3_ (10 nM). The upper panels show the fraction of VDR located to the nucleus and the cytosol in grey and black columns, respectively, as determined by the density of the VDR bands from the Western blots. The lower panels show one representative Western blotting analysis of VDR and GAPDH (loading control) from the cytosolic (C) and nuclear (N) fraction of CD4^+^ T cells activated in the absence (-) or presence (+) of 1,25(OH)_2_D_3_ and RA as indicated. **(B)** Mean ± SD (VDR^WT^ n = 14; VDR^R80W^ n = 1 repeated 5 times; *p < 0.05). **(C, D)** Mean ± SD (VDR^WT^ n = 8; VDR^R80W^ n = 1 repeated 3 times; *p < 0.05). **(E)** Relative mRNA expression of CYP24A1 in VDR^WT^ (black circles) and VDR^R80W^ (white squares) CD4^+^ T cells activated in the absence (-) or presence (+) of RA (1 µM) and 1,25(OH)_2_D_3_ (10 nM). The expression levels are given as fold change normalized to the average expression level of CYP24A1 in control cells activated in the absence of RA and 1,25(OH)_2_D_3_. Mean ± SD (VDR^WT^ n = 6; VDR^R80W^ n = 1 repeated twice; *p < 0.05).

Previous studies have shown that binding of 1,25(OH)_2_D_3_ up-regulates the VDR by protecting it from proteasomal degradation in human T cells and keratinocytes (20, 24). To determine whether binding of 1,25(OH)_2_D_3_ was compromised in VDR^R80W^, we determined the efficiency of 1,25(OH)_2_D_3_ to up-regulate the VDR. We activated naïve CD4^+^ T cells from the patient and controls in the absence or presence of 10 nM 1,25(OH)_2_D_3_ and determined the amount of VDR by Western blotting analysis. As previously shown, 1,25(OH)_2_D_3_ stabilized the VDR leading to a 2-3 fold up-regulation of the VDR in control cells. We found a similar 1,25(OH)_2_D_3_-induced VDR stabilization and up-regulation in the cells of the patient indicating that binding of 1,25(OH)_2_D_3_ was intact in VDR^R80W^ (**Figure 3A**).

It was previously demonstrated that the VDR is distributed to both the cytosol and the nucleus in the absence of 1,25(OH)_2_D_3_, and that binding of 1,25(OH)_2_D_3_ shifts the localization of the VDR in favor of the nucleus (20, 25). To determine whether 1,25(OH)_2_D_3_ induced a shift in the subcellular localization of VDR^R80W^, we activated CD4^+^ T cells from the patient and controls in the absence or presence of 10 nM 1,25(OH)_2_D_3_ and determined the fraction of VDR located to the cytosol and the nucleus by Western blotting analysis. Whereas the majority of VDR^WT^ localized to the nucleus in T cell activated in the absence of 1,25(OH)_2_D_3_, the vast majority of VDR^R80W^ localized to the cytoplasm (**Figure 3B**). However, as for VDR^WT^, 1,25(OH)_2_D_3_ clearly induced significant nuclear translocation of VDR^R80W^ confirming intact binding of 1,25(OH)_2_D_3_ to VDR^R80W^.

The subcellular localization of the VDR is a consequence of its binding to ligand, RXR and DNA (7, 9, 25). Our findings described above indicated that ligand binding was intact in VDR^R80W^. Therefore the disturbed subcellular localization of VDR^R80W^ must be caused either by affected RXR binding, affected DNA binding or a combination of the two. Previous studies have suggested that RA can inhibit VDR-RXR heterodimer formation (9, 10). As VDR-RXR heterodimer formation is involved in the 1,25(OH)_2_D_3_-induced translocation of the VDR to the nucleus (7, 9, 25), and as binding of 1,25(OH)_2_D_3_ was unaffected in VDR^R80W^, we could directly determine if VDR-RXR heterodimer formation was intact in the cells of the patient by analyzing the effect of RA on 1,25(OH)_2_D_3_-induced translocation of the VDR to the nucleus. Consequently, we activated naïve CD4^+^ T cells from the patient and controls in the absence or presence of 1,25(OH)_2_D_3_ and RA and determined the fraction of VDR located in the cytosol and the nucleus by Western blotting analysis. In both patient and control cells, we found that RA alone did not affect VDR localization, that 1,25(OH)_2_D_3_ alone induced translocation of the VDR to the nucleus, and most interestingly that RA significantly inhibited the 1,25(OH)_2_D_3_-induced translocation of the VDR to the nucleus (**Figures 3C, D**). These data indicated that VDR-RXR heterodimer formation was intact in the cells of the patient and that the dramatically affected subcellular location of VDR^R80W^ was caused by the affected ability of VDR^R80W^ to bind DNA.

To study whether RA-mediated suppression of VDR translocation to the nucleus was associated with impaired vitamin D signaling, we activated CD4^+^ T cells from the patient and controls in the absence or presence of RA and 1,25(OH)_2_D_3_ and measured mRNA expression of CYP24A1 by RT-qPCR. RA alone did not affect the expression, whereas 1,25(OH)_2_D_3_ alone strongly up-regulated CYP24A1 expression in control cells (**Figure 3E**). Interestingly, RA significantly inhibited 1,25(OH)_2_D_3_-induced up-regulation of CYP24A1 by approximately 35% in control cells. As expected, neither 1,25(OH)_2_D_3_ nor RA affected CYP24A1 in T cells from the patient (**Figure 3E**).

### Reduced Responsiveness to 1,25(OH)_2_D_3_ in T Cells From Heterozygous Family Members

**Figure 4:**
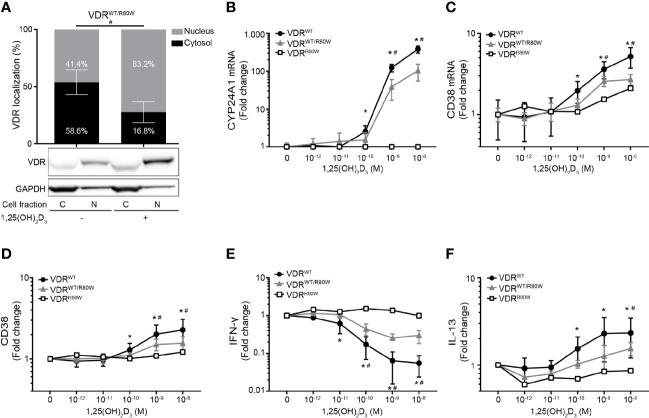
Reduced responsiveness to 1,25(OH)_2_D_3_ in T cells from heterozygous family members **(A)** Subcellular localization of the VDR in VDR^WT/R80W^ heterozygous CD4^+^ T cells activated in the absence (-) or presence (+) of 1,25(OH)_2_D_3_ (10 nM). The upper panel shows the fraction of VDR located to the nucleus and the cytosol in grey and black columns, respectively, as determined by the density of the VDR bands from the Western blotting analysis. The lower panels show one representative Western blotting analysis out of four independent experiments of VDR and GAPDH (loading control) from the cytosolic (C) and nuclear (N) fraction. Mean ± SD (VDR^WT/R80W^ n = 4; ^#^p < 0.05). **(B)** CYP24A1 and **(C)** CD38 mRNA expression, **(D)** CD38 cell surface expression, **(E)** IFN-γ and **(F)** IL-13 production in VDR^WT^ (black circles), VDR^WT/R80W^ (gray triangles) and VDR^R80W^ (white squares) CD4^+^ T cells activated in the presence of the indicated concentration of 1,25(OH)_2_D_3_. The expression levels are given as fold change normalized to the average expression level of the given molecule in cells activated in the absence of 1,25(OH)_2_D_3_ in each group. Mean ± SD (VDR^WT^ n = 13; VDR^WT/R80W^ n = 4; VDR^R80W^ n = 1; *VDR^WT^ p < 0.05 compared to VDR^WT^ in the absence of 1,25(OH)_2_D_3_, ^#^VDR^WT/R80W^ p < 0.05 compared to VDR^WT/R80W^ in the absence of 1,25(OH)_2_D_3_).

To establish whether VDR^WT^ and VDR^R80W^ were co-expressed in T cells of the heterozygous family members, we activated their CD4^+^ T cells in the absence or presence of 10 nM 1,25(OH)_2_D_3_ and determined the fraction of VDR located to the cytosol and the nucleus by Western blotting analysis. We found that the VDR in the heterozygous family members was located in a pattern in between the controls and the patient with approximately 59% of the VDR in the cytosol and 41% in the nucleus in the absence of 1,25(OH)_2_D_3_ (**Figure 4A**). To determine the consequences of the VDR^R80W^ mutation in T cell function, we activated CD4^+^ T cells from the patient, the heterozygous family members and controls in the presence of 0 – 10 nM 1,25(OH)_2_D_3_ and determined the expression of some of the genes and gene products classically known to be regulated by 1,25(OH)_2_D_3_. Whereas the patient was completely unresponsive to 1,25(OH)_2_D_3_, we found that the 1,25(OH)_2_D_3_ responsiveness was reduced by ~ 50% in T cells from the heterozygous family members (**Figures 4B–F**). This indicated that the VDR^WT^ and the VDR^R80W^ were expressed at similar levels in T cells of the heterozygous family members and that the expression level of VDR^WT^ determined 1,25(OH)_2_D_3_ responsiveness.

### The R80W Mutation Abolishes 1,25(OH)_2_D_3_-Mediated Suppression of RA-Induced Gene Up-Regulation

**Figure 5:**
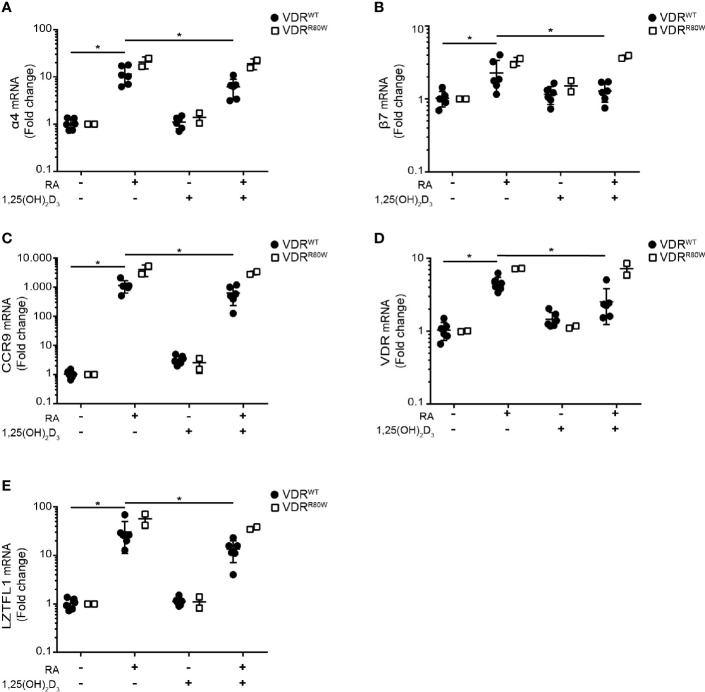
The R80W mutation abolishes 1,25(OH)_2_D_3_-mediated suppression of RA-induced gene up-regulation Relative mRNA expression of **(A)** α4, **(B)** β7, **(C)** CCR9, **(D)** VDR and **(E)** LZTFL1 in VDR^WT^ (black circles) and VDR^R80W^ (white squares) CD4^+^ T cells activated in the absence (-) or presence (+) of RA (1 µM) and 1,25(OH)_2_D_3_ (10 nM). The expression levels are given as fold change normalized to the average expression level of the target gene in each group activated in the absence of RA and 1,25(OH)_2_D_3_. Mean ± SD (VDR^WT^ n = 6; VDR^R80W^ n = 1 repeated twice; *p < 0.05).

RA enhances expression of integrin α4β7 and CCR9 on T cells upon activation and imprints them with gut tropism (1, 26–28). Interestingly, it has been reported that 1,25(OH)_2_D_3_ suppresses the RA-induced expression of α4β7 and CCR9 (14). To study the role of the VDR in 1,25(OH)_2_D_3_-mediated suppression of RA-induced expression of α4β7 and CCR9 in T cells, we activated CD4^+^ T cells from the patient and controls in the absence or presence of RA and 1,25(OH)_2_D_3_ and measured mRNA expression of integrin subunits α4 and β7 and CCR9 by RT-qPCR. RA up-regulated α4, β7 and CCR9 in control cells, whereas 1,25(OH)_2_D_3_ alone did not affect their expression **(Figures 5A–C)**. However, 1,25(OH)_2_D_3_ significantly inhibited RA-induced up-regulation of α4, β7 and CCR9 in control cells but not in T cells from the patient. In the absence of 1,25(OH)_2_D_3_, the RA-induced up-regulation of α4, β7 and CCR9 was slightly enhanced in T cells of the patient compared to control cells (**Figures 5A–C**).

To determine whether VDR-mediated suppression of RA-induced genes affected other genes than the genes involved in gut tropism, we searched for additional genes not affected by vitamin D but up-regulated by vitamin A in T cells. It has been shown that RA up-regulates VDR gene expression (29, 30), whereas 1,25(OH)_2_D_3_ does not affect VDR gene expression in T cells (20). Likewise, the leucine zipper transcription factor-like 1 (LZTFL1) gene is unaffected by vitamin D but up-regulated by vitamin A in T cells (31). We found exactly the same pattern of gene regulation for VDR and LZTFL1 as for α4, β7 and CCR9. RA alone up-regulated VDR and LZTFL1 expression in both patient and control cells, 1,25(OH)_2_D_3_ alone did not affect VDR and LZTFL1 expression, and 1,25(OH)_2_D_3_ significantly inhibited RA-induced VDR and LZTFL1 up-regulation in control cells but not in cells from the patient (**Figures 5D, E**).

## Discussion

In this study, we describe a new mutation in the VDR and the consequences of this mutation for vitamin D and A signaling in T cells from a family carrying the mutation. The R80W mutation is located to the C terminal part of the second zinc finger in the DBD of the VDR. When the VDR binds DNA, R80 forms hydrogen bonds to the backbone of DNA (22), and it would be predicted that the R80W mutation therefore most likely affected DNA binding. This was supported by our *in silico* analysis that demonstrated that the hydrogen bonds normally formed between R80 and the DNA backbone were not allowed in VDR^R80W^. Furthermore, the *in silico* analysis indicated that the R80W mutation only affected binding to DNA and that other areas of VDR^R80W^ were unaffected. In accordance, we found that the transcriptional activity of VDR^R80W^ was severely affected, whereas binding of 1,25(OH)_2_D_3_ and RXR was intact as measured by normal 1,25(OH)_2_D_3_-induced up-regulation and translocation to the nucleus of the VDR^R80W^ (20, 24, 25).

A different mutation, which caused substitution of a glutamine (Q) for the R at position 80 in the VDR (R80Q), has previously been described in three apparently unrelated families originating in North Africa (32, 33). In agreement with our observations, the R80Q mutation did not affect binding of 1,25(OH)_2_D_3_ but affected nuclear localization and DNA binding of the 1,25(OH)_2_D_3_-VDR complex. In the present study, we found that the R80W mutation strongly affected the subcellular localization of the VDR^R80W^. These observations demonstrate that the DNA binding ability of the VDR influences the subcellular localization of the VDR in both the absence and presence of 1,25(OH)_2_D_3_ and they suggest that non-liganded VDR normally binds DNA.

Although the heterozygous family members did not have clinical signs of HVDRR, the responsiveness of their T cells to 1,25(OH)_2_D_3_ was reduced by approximately 50%. This indicated that VDR^WT^ and VDR^R80W^ were expressed to the same extent in T cells of the heterozygous family members. If 1,25(OH)_2_D_3_ was the limiting factor in 1,25(OH)_2_D_3_ responsiveness in T cells, it would be expected that doubling the concentration of 1,25(OH)_2_D_3_ should restore 1,25(OH)_2_D_3_ responsiveness in the heterozygous family members. However, even a 10-fold increase in 1,25(OH)_2_D_3_ did not fully restore the responsiveness, which strongly supported that the expression level of the VDR^WT^ determines 1,25(OH)_2_D_3_ responsiveness in T cells as previously suggested for other types of cells (24). Although increased concentrations of 1,25(OH)_2_D_3_ did not fully restore 1,25(OH)_2_D_3_ responsiveness in the T cells of the heterozygous family members, it did increase the responsiveness. From these observations, it may be suggested that, although homozygous HVDRR patients with a mutations in the DBD of the VDR do not benefit from vitamin D supplementation, heterozygous carriers of these mutations might actually benefit from high-dose vitamin D supplementation.

Previous studies have reported that vitamin A antagonizes the calcium response to vitamin D in man and rat (34, 35). However, the mechanism behind this RA-mediated suppression of vitamin D signaling is unknown. Here we demonstrated that RA inhibits 1,25(OH)_2_D_3_-induced translocation of the VDR to the nucleus and 1,25(OH)_2_D_3_-induced up-regulation of CYP24A1. Together with the notion that the VDR expression level determines vitamin D responsiveness, it might be suggested that the suppression of VDR translocation to the nucleus mediated by RA results in the decreased 1,25(OH)_2_D_3_-induced transcriptional activity of the VDR. Other mechanisms, such as competition between RAR and VDR for binding to VDRE, cannot be excluded and require further experiments to be determined.

We found that 1,25(OH)_2_D_3_ inhibited RA-induced gene transcription in control cells but not in cells from the HVDRR patient. Furthermore, RA-induced gene transcription in the absence of 1,25(OH)_2_D_3_ was slightly increased in cells of the patient compared to control cells. From this, we suggest that the VDR can affect RA-induced gene regulation in both the absence and presence of 1,25(OH)_2_D_3_. Furthermore, this suppressive effect is most likely dependent on the ability of the VDR to bind DNA. This is in accordance with previous studies that have suggested that non-liganded VDR might bind DNA in association with the co-repressors (9, 36) and that VDR-RXR complexes can bind and block RARE (11, 12). Other mechanisms responsible for the suppressive effect of 1,25(OH)_2_D_3_ on RA-induced gene transcription may be suggested. Thus, one study found that vitamin D suppressed the transcriptional activity of the androgen receptor, and it was suggested that this might be caused by direct binding and inhibition of the androgen receptor by the liganded VDR (37).

Due to the lack of 1,25(OH)_2_D_3_-mediated inhibition of RA-induced gene transcription in the T cells of the patient in the present study, it might be suggested that the patient has a functional hypervitaminosis A. Accordingly, despite normal serum levels of calcium, phosphate, alkaline phosphatase and parathyroid hormone, the patient’s main complains were bone pains and hair loss, classical signs of hypervitaminosis A (38, 39). Further studies are required to test this hypothesis.

In conclusion, by characterization of a novel mutation in the DBD of the VDR, we show that the expression level of wild-type VDR determines 1,25(OH)_2_D_3_ responsiveness in T cells. We show that vitamin D-mediated suppression of vitamin A-induced gene regulation is dependent on R80 and thereby most probably the ability of VDR to bind DNA. Furthermore, we demonstrate that vitamin A inhibits 1,25(OH)_2_D_3_-induced translocation of the VDR to the nucleus and 1,25(OH)_2_D_3_-induced up-regulation of CYP24A1. Our study suggests that vitamin D deficiency might not only result in decreased vitamin D signaling but also in increased vitamin A signaling. Further studies are required to elucidate the mechanisms and physiological role of this intriguing interplay between vitamin D and A signaling.

## Data Availability Statement

The RNAseq data presented in this article are not readily available due to patient confidentiality. Further inquiries can be directed to the corresponding author.

## Ethics Statement

The studies involving human participants were reviewed and approved by The Committees of Biomedical Research Ethics for the Capital Region in Denmark (H-170409222). The patients/participants provided their written informed consent to participate in this study. Written informed consent was obtained from the individual(s) for the publication of any potentially identifiable images or data included in this article.

## Author Contributions

CG, MK-W and FA-J conceived the study and designed experiments. AA-O and SB performed *in silico* experiments and analysis. NK contributed to the genetic experiments and analysis. TB contributed to the RNA sequencing experiments and analysis. DL and AR contributed to the flow cytometric experiments and analysis. EG and KO contributed to the recruitment of the test subjects. AW, NØ and CB assisted with the experimental design and data interpretation. CG, FA-J and MK-W wrote the manuscript with input from all authors. All authors contributed to the article and approved the submitted version.

## Funding

This study was supported by The Danish Council for Independent Research grant number 8020-00066B.

## Conflict of Interest

The authors declare that the research was conducted in the absence of any commercial or financial relationships that could be construed as a potential conflict of interest.

## References

[1] Mora JR Iwata M von Andrian UH. Vitamin Effects on the Immune System: Vitamins A and D Take Centre Stage. Nat Rev Immunol (2008) 8(9):685–98. 10.1038/nri2378 PMC2906676 19172691

[2] Kongsbak M Levring TB Geisler C von Essen MR. The Vitamin D Receptor and T Cell Function. Front Immunol (2013) 4:148. 10.3389/fimmu.2013.00148 23785369 PMC3684798

[3] Kongsbak M von Essen MR Levring TB Schjerling P Woetmann A Odum N. Vitamin D-binding Protein Controls T Cell Responses to Vitamin D. BMC Immunol (2014) 15:1–13. 10.1186/s12865-014-0035-2 25230725 PMC4177161

[4] Rode AKO Kongsbak M Hansen MM Lopez DV Levring TB Woetmann A. Vitamin D Counteracts Mycobacterium Tuberculosis-Induced Cathelicidin Downregulation in Dendritic Cells and Allows Th1 Differentiation and IFNgamma Secretion. Front Immunol (2017) 8:656. 10.3389/fimmu.2017.00656 28620394 PMC5450038

[5] Mangelsdorf DJ Thummel C Beato M Herrlich P Schutz G Umesono K. The Nuclear Receptor Superfamily: The Second Decade. Cell (1995) 83(6):835–9. 10.1016/0092-8674(95)90199-X PMC6159888 8521507

[6] Bain DL Heneghan AF Connaghan-Jones KD Miura MT. Nuclear Receptor Structure: Implications for Function. Annu Rev Physiol (2007) 69:201–20. 10.1146/annurev.physiol.69.031905.160308 17137423

[7] Haussler MR Whitfield GK Kaneko I Haussler CA Hsieh D Hsieh JC. Molecular Mechanisms of Vitamin D Action. Calcif Tissue Int (2013) 92(2):77–98. 10.1007/s00223-012-9619-0 22782502

[8] Xu L Glass CK Rosenfeld MG. Coactivator and Corepressor Complexes in Nuclear Receptor Function. Curr Opin Genet Dev (1999) 9(2):140–7. 10.1016/S0959-437X(99)80021-5 10322133

[9] Haussler MR Haussler CA Jurutka PW Thompson PD Hsieh JC Remus LS. The Vitamin D Hormone and its Nuclear Receptor: Molecular Actions and Disease States. J Endocrinol (1997) 154 Suppl S57–73:1–15. 9379138

[10] Thompson PD Jurutka PW Haussler CA Whitfield GK Haussler MR. Heterodimeric DNA Binding by the Vitamin D Receptor and Retinoid X Receptors is Enhanced by 1,25-Dihydroxyvitamin D3 and Inhibited by 9-Cis-Retinoic Acid. Evidence for Allosteric Receptor Interactions. J Biol Chem (1998) 273(14):8483–91. 10.1074/jbc.273.14.8483 9525962

[11] Garcia-Villalba P Jimenez-Lara AM Aranda A. Vitamin D Interferes With Transactivation of the Growth Hormone Gene by Thyroid Hormone and Retinoic Acid. Mol Cell Biol (1996) 16(1):318–27. 10.1128/MCB.16.1.318 PMC231006 8524311

[12] Jimenez-Lara AM Aranda A. The Vitamin D Receptor Binds in a Transcriptionally Inactive Form and Without a Defined Polarity on a Retinoic Acid Response Element. FASEB J (1999) 13(9):1073–81. 10.1096/fasebj.13.9.1073 10336890

[13] Bastie JN Balitrand N Guidez F Guillemot I Larghero J Calabresse C. 1 Alpha,25-Dihydroxyvitamin D3 Transrepresses Retinoic Acid Transcriptional Activity Via Vitamin D Receptor in Myeloid Cells. Mol Endocrinol (2004) 18(11):2685–99. 10.1210/me.2003-0412 15284334

[14] Sigmundsdottir H Pan J Debes GF Alt C Habtezion A Soler D. Dcs Metabolize Sunlight-Induced Vitamin D3 to ‘Program’ T Cell Attraction to the Epidermal Chemokine CCL27. Nat Immunol (2007) 8(3):285–93. 10.1038/ni1433 17259988

[15] Feldman D Malloy J. Mutations in the Vitamin D Receptor and Hereditary Vitamin D-resistant Rickets. Bonekey Rep (2014) 3:510. 10.1038/bonekey.2014.5 24818002 PMC4015455

[16] Malloy PJ Tasic V Taha D Tutunculer F Ying GS Yin LK. Vitamin D Receptor Mutations in Patients With Hereditary 1,25-Dihydroxyvitamin D-resistant Rickets. Mol Genet Metab (2014) 111(1):33–40. 10.1016/j.ymgme.2013.10.014 24246681 PMC3933290

[17] Webb B Sali A. Comparative Protein Structure Modeling Using Modeller. Curr Protoc Bioinf (2016) 54:5.6.1–5.6.37. 10.1002/cpbi.3 PMC5031415 27322406

[18] Bray NL Pimentel H Melsted P Pachter L. Near-Optimal Probabilistic RNA-seq Quantification. Nat Biotechnol (2016) 34(5):525–7. 10.1038/nbt.3519 27043002

[19] Vadivel CK Gluud M Torres-Rusillo S Boding L Willerslev-Olsen A Buus TB. Jak3 Is Expressed in the Nucleus of Malignant T Cells in Cutaneous T Cell Lymphoma (Ctcl). Cancers (Basel) (2021) 13(2). 10.3390/cancers13020280 PMC7828698 33466582

[20] Kongsbak M von Essen MR Boding L Levring TB Schjerling P Lauritsen JP. Vitamin D Up-Regulates the Vitamin D Receptor by Protecting it From Proteasomal Degradation in Human CD4+ T Cells. PloS One (2014) 9(5):e96695. 10.1371/journal.pone.0096695 24792400 PMC4008591

[21] Levring TB Kongsbak-Wismann M Rode AKO Al-Jaberi FAH Lopez DV Met O. Tumor Necrosis Factor Induces Rapid Down-Regulation of TXNIP in Human T Cells. Sci Rep (2019) 9(1):16725. 10.1038/s41598-019-53234-x 31723203 PMC6853882

[22] Shaffer PL Gewirth DT. Structural Basis of VDR-DNA Interactions on Direct Repeat Response Elements. EMBO J (2002) 21(9):2242–52. 10.1093/emboj/21.9.2242 PMC125986 11980721

[23] von Essen MR Kongsbak M Schjerling P Olgaard K Odum N Geisler C. Vitamin D Controls T Cell Antigen Receptor Signaling and Activation of Human T Cells. Nat Immunol (2010) 11(4):344–9. 10.1038/ni.1851 20208539

[24] Li XY Boudjelal M Xiao JH Peng ZH Asuru A Kang S. 1,25-Dihydroxyvitamin D3 Increases Nuclear Vitamin D3 Receptors by Blocking Ubiquitin/Proteasome-Mediated Degradation in Human Skin. Mol Endocrinol (1999) 13(10):1686–94. 10.1210/mend.13.10.0362 10517670

[25] Prufer K Racz A Lin GC Barsony J. Dimerization With Retinoid X Receptors Promotes Nuclear Localization and Subnuclear Targeting of Vitamin D Receptors. J Biol Chem (2000) 275(52):41114–23. 10.1074/jbc.M003791200 11001945

[26] Iwata M Hirakiyama A Eshima Y Kagechika H Kato C Song SY. Retinoic Acid Imprints Gut-Homing Specificity on T Cells. Immunity (2004) 21(4):527–38. 10.1016/j.immuni.2004.08.011 15485630

[27] Kang SG Park J Cho JY Ulrich B Kim CH. Complementary Roles of Retinoic Acid and TGF-beta1 in Coordinated Expression of Mucosal Integrins by T Cells. Mucosal Immunol (2011) 4(1):66–82. 10.1038/mi.2010.42 20664575 PMC3012787

[28] Koenecke C Prinz I Bubke A Schreder A Lee CW Pabst O. Shift of Graft-Versus-Host-Disease Target Organ Tropism by Dietary Vitamin a. PloS One (2012) 7(5):e38252. 10.1371/journal.pone.0038252 22666498 PMC3364223

[29] Miyamoto K Kesterson RA Yamamoto H Taketani Y Nishiwaki E Tatsumi S. Structural Organization of the Human Vitamin D Receptor Chromosomal Gene and its Promoter. Mol Endocrinol (1997) 11(8):1165–79. 10.1210/mend.11.8.9951 9212063

[30] Janik S Nowak U Laszkiewicz A Satyr A Majkowski M Marchwicka A. Diverse Regulation of Vitamin D Receptor Gene Expression by 1,25-Dihydroxyvitamin D and ATRA in Murine and Human Blood Cells at Early Stages of Their Differentiation. Int J Mol Sci (2017) 18(6):1–17. 10.3390/ijms18061323 PMC5486144 28635660

[31] Jiang H Promchan K Lin BR Lockett S Chen D Marshall H. Lztfl1 Upregulated by All-Trans Retinoic Acid During CD4+ T Cell Activation Enhances IL-5 Production. J Immunol (2016) 196(3):1081–90. 10.4049/jimmunol.1500719 PMC4724573 26700766

[32] Sone T Marx SJ Liberman UA Pike JW. A Unique Point Mutation in the Human Vitamin D Receptor Chromosomal Gene Confers Hereditary Resistance to 1,25-Dihydroxyvitamin D3. Mol Endocrinol (1990) 4(4):623–31. 10.1210/mend-4-4-623 2177843

[33] Malloy PJ Weisman Y Feldman D. Hereditary 1 Alpha,25-Dihydroxyvitamin D-resistant Rickets Resulting From a Mutation in the Vitamin D Receptor Deoxyribonucleic Acid-Binding Domain. J Clin Endocrinol Metab (1994) 78(2):313–6. 10.1210/jcem.78.2.8106618 8106618

[34] Rohde CM DeLuca HF. All-Trans Retinoic Acid Antagonizes the Action of Calciferol and its Active Metabolite, 1,25-Dihydroxycholecalciferol, in Rats. J Nutr (2005) 135(7):1647–52. 10.1093/jn/135.7.1647 15987844

[35] Johansson S Melhus H. Vitamin A Antagonizes Calcium Response to Vitamin D in Man. J Bone Miner Res (2001) 16(10):1899–905. 10.1359/jbmr.2001.16.10.1899 11585356

[36] Puccetti E Obradovic D Beissert T Bianchini A Washburn B Chiaradonna F. AML-Associated Translocation Products Block Vitamin D(3)-induced Differentiation by Sequestering the Vitamin D(3) Receptor. Cancer Res (2002) 62(23):7050–8. 12460926

[37] Kawata H Kamiakito T Takayashiki N Tanaka A. Vitamin D3 Suppresses the Androgen-Stimulated Growth of Mouse Mammary Carcinoma SC-3 Cells by Transcriptional Repression of Fibroblast Growth Factor 8. J Cell Physiol (2006) 207(3):793–9. 10.1002/jcp.20618 16508948

[38] Baineni R Gulati R Delhi CK. Vitamin A Toxicity Presenting as Bone Pain. Arch Dis Child (2017) 102(6):556–8. 10.1136/archdischild-2016-310631 27272974

[39] Rutkowski M Grzegorczyk K. Adverse Effects of Antioxidative Vitamins. Int J Occup Med Environ Health (2012) 25(2):105–21. 10.2478/s13382-012-0022-x 22528540

