# Supplementary figures and images for: Impaired Vitamin D Signaling in T Cells From a Family With Hereditary Vitamin D Resistant Rickets
Source: Front Immunol. 2021 May 19;12:684015. doi: 10.3389/fimmu.2021.684015 (PMC8170129; doi:10.3389/fimmu.2021.684015)

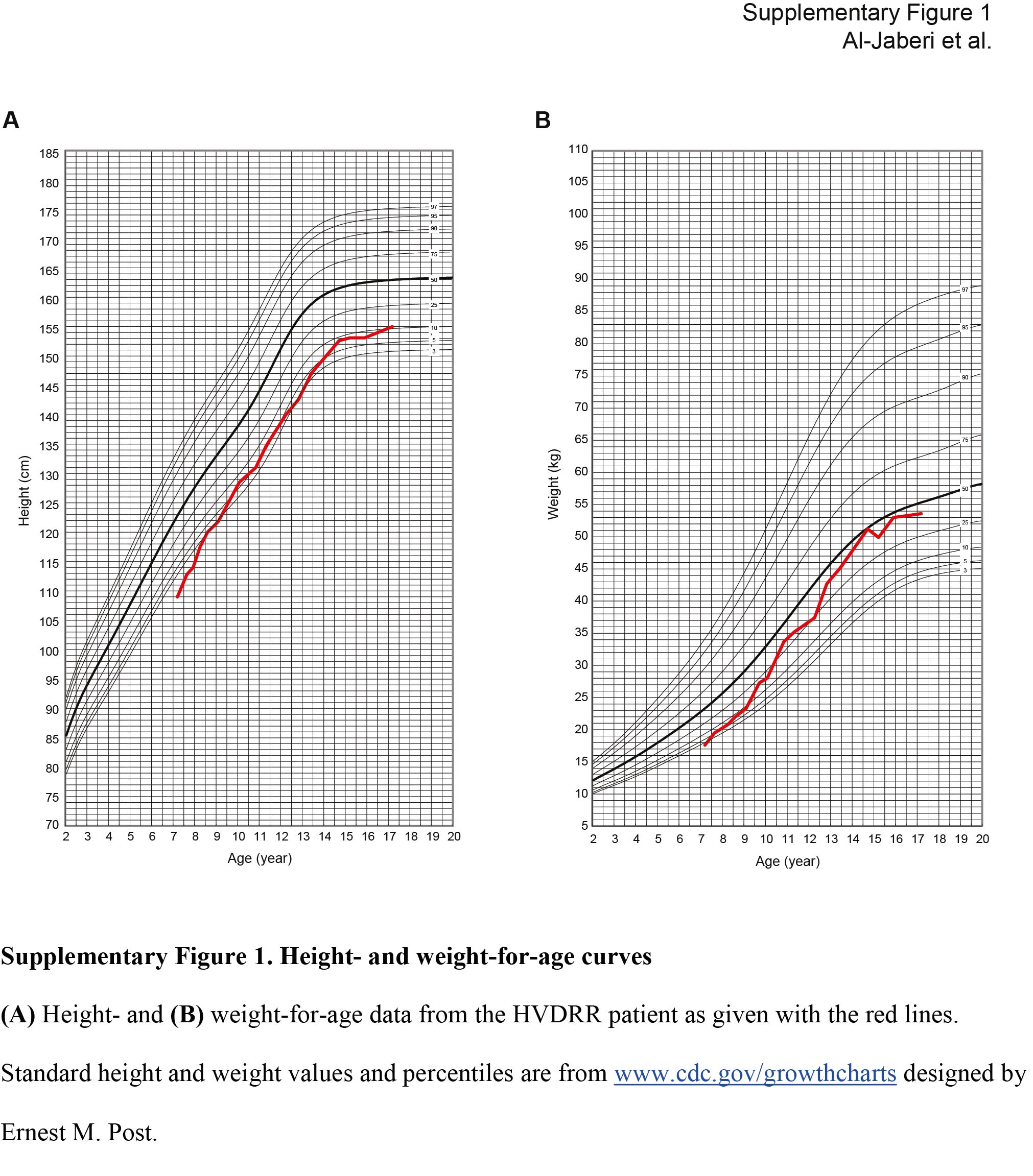

Supplement: Supplementary file 1 [file Image_1.jpeg]

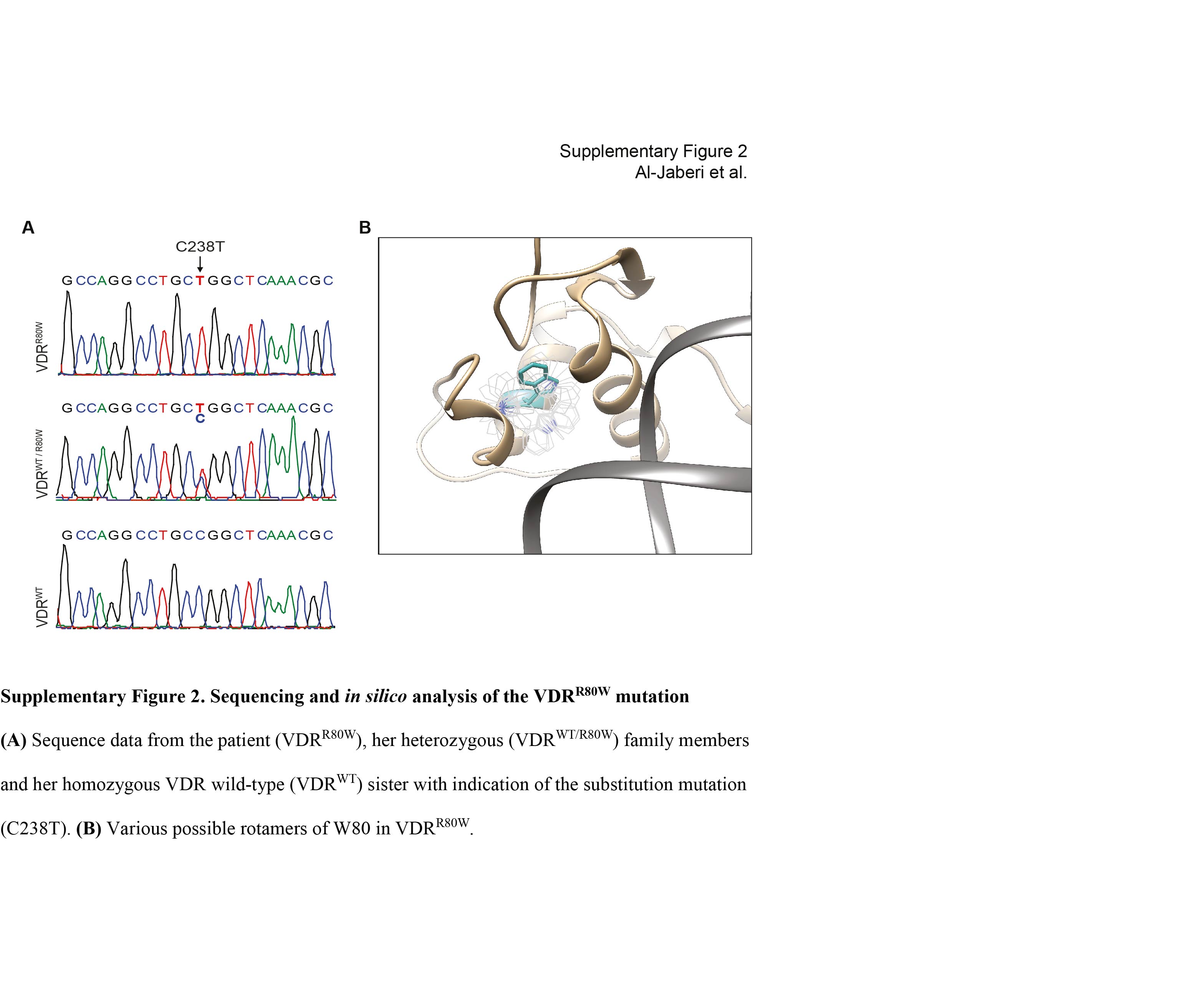

Supplement: Supplementary file 2 [file Image_2.jpeg]
